# Atypical Response of B-1 Cells to BCR Ligation: A Speculative Model

**DOI:** 10.3389/fimmu.2013.00457

**Published:** 2013-12-16

**Authors:** Nichol E. Holodick, Thomas L. Rothstein

**Affiliations:** ^1^Center for Oncology and Cell Biology, The Feinstein Institute for Medical Research, Manhasset, NY, USA

**Keywords:** B cells, signal transduction, protein kinases/phosphatases, rodent

## Abstract

Peritoneal B-1a cells manifest unusual signaling characteristics that distinguish them from splenic B-2 cells. These include the failure of BCR engagement to trigger NF-κB activation and DNA replication. Despite extensive study, a clear explanation for these characteristics has not emerged. Here we aim to develop a unified paradigm based on previous reports and recent results, which proposes a central role for phosphatase activity. We hypothesize B-1a cells are unable to induce NF-κB or proliferate after BCR cross-linking due to increased phosphatase abundance or activity. This phosphatase abundance and/or activity may be the result of unique B-1a cell characteristics such as increased levels of HSP70 and/or constitutive secretion of IL-10. We speculate phosphatase activity cannot be overcome by BCR ligation alone due to insufficient Vav protein expression, which does not allow for proper production of reactive oxygen species, which inhibit phosphatases. Furthermore, constitutively active Lyn also plays a negative regulatory role in B-1a. We expect that a new focus on phosphatase activity and its suppression will be revealing for BCR signal transduction in B-1 cells.

## B-1 Cell Overview

### B-1 cell characteristics

B-1a cells are set apart from conventional B2 cells based on phenotypic and functional differences. B-1a cells are phenotypically characterized by the following cell surface markers: B220^lo^, CD5^+^, immunoglobulin (Ig) (sIg) M^hi^, sIgD^lo^, Mac-1^+^, CD23^−^, and CD43^+^ (1, 2). In mice the largest proportion of B-1a cells are found in the peritoneal cavity with a small proportion but approximately equal sized population residing in the spleen (3, 4). The B-1a cell population originates during fetal life and persists throughout adult life by their ability to self-renew, meaning new B-1a cells are generated by mitosis of mature surface Ig-expressing B-1a cells. This process is regulated in a feedback fashion (5, 6). B-1a cell self-renewal is unlike development of B-2 cells, wherein mature cells derive from surface Ig-negative progenitors. Recently early appearing B-1a cells were shown to represent a separate lineage derived from a unique progenitor found both in the fetal liver and bone marrow that does not give rise to B-2 cells (7).

B-1a cells exhibit a number of functional characteristics different from conventional B-2 cells. B-1a cells spontaneously secrete IgM, which is often referred to as natural antibody and accumulates as the bulk of resting or non-immune IgM. Ig secreted by unstimulated B-1a cells varies less from germline than Ig secreted by B-2 cells, which is because B-1a immunoglobulin undergoes minimal if any somatic hypermutation and possesses little N-region addition (8–10). In addition, B-1a cells are repertoire skewed as evidenced by biased variable heavy chain (VH) gene usage in favor of V_H_11 and V_H_12 (9–13). This skewed, germline-like repertoire contains both antimicrobial and autoreactive specificities. B-1a cell-derived natural IgM has been shown to be essential for: (1) anti-microbial protection, through initial serological control of bacterial and viral infections (14–16), and (2) housekeeping homeostasis, by aiding in disposal of autoantigens through removal of apoptotic cell debris (17–19). In addition, housekeeping natural antibodies assist in elimination of toxic molecules such as oxidized low density lipoprotein (oxLDL), in particular by antibodies bearing the T15 idiotype, which helps control the inflammatory process leading to atherosclerotic plaques (20). These diverse functions may be facilitated by the characteristic polyreactivity of B-1a cell Ig.

Beyond spontaneous secretion of natural IgM antibody, B-1a cells express other distinct functions not shared by resting conventional B-2 cells. B-1a cells present antigen more potently than conventional B-2 cells, a property that has been attributed to constitutive expression of the co-stimulatory molecules B7.1 and B7.2 (21–23). Further, B-1a cells have been shown to induce pro-inflammatory Th17 cell differentiation and to generate immunosuppressive IL-10 (23, 24). Thus, in addition to antibody production, B-1a cells can influence other elements of the immune system in both positive and negative ways.

B-1a cells express unique signaling and proliferative characteristics, which seem in some ways hyperresponsive in comparison to B-2 cells but in other ways hyporesponsive. B-1a cells display constitutive expression of activated signaling mediators including ERK, NF-AT, and STAT3 (25, 26), which in B-2 cells require stimulation for activated expression (27). B-1a cells have also been shown to proliferate in response to treatment with phorbol ester as a single agent, in contrast to B-2 cells, which only respond to phorbol myristate acetate or phorbol dibutyrate in conjunction with a calcium ionophore (28). PMA responsiveness in B-1a cells is associated with rapid induction of cyclin D2 and activation of RB-phosphorylating cyclin D3-cdk4 complexes, neither of which occur in PMA-treated B-2 cells (29, 30). However, despite activated signaling mediators at rest and despite hyperresponsiveness to PMA, BCR signaling fails in B-1a cells – NF-κB is not induced nor is proliferation stimulated.

### BCR signaling in B-1a cells

Despite the failure of BCR engagement to induce NF-κB activation in B-1a cells (31, 32), stimulation with LPS or PMA succeeds just as in B-2 cells (31), suggesting that key components involved in the pathway proximal to induction of this transcription factor are not lacking. Several previous studies have sought to determine why B-1a cells have an attenuated response to BCR cross-linking as compared to B-2 cells when the basic NF-κB machinery appears intact. The negative regulatory receptor CD5 and the tyrosine phosphatase SHP-1 were reported to play a role in the failure of B-1a cells to respond to BCR ligation. It was demonstrated B-1a cells from CD5 deficient mice could respond to BCR ligation, and SHP-1 was shown to be constitutively associated with the BCR in a CD5-dependent manner (33). The important regulatory role SHP-1 plays in B-1a cell development was presented in a separate study, which demonstrated an increase in B-1a cell number in the absence of SHP-1 and the negative regulation it provides; however, there was no change in the ability of SHP-1 deficient B-1a cells to proliferate in response to BCR crosslinking (34). Despite the initial clear results demonstrating CD5/SHP-1 negatively regulates BCR signaling in B-1a cells, it was later shown both B-1a (CD5^+^) and B-1b (CD5^−^) cells fail to respond to BCR ligation (35), raising questions about the role of CD5 and associated molecules. These results suggest some other element is responsible for the lack of B-1a cell responsiveness to BCR engagement, whereas the extent of CD5 involvement remains uncertain.

The src family kinase Lck, which characterizes T cells, was reported to be unexpectedly expressed in B-1a cells and responsible for defective NF-κB activation in response to BCR ligation (36). Dal Porto et al. reported peritoneal B-1a cells express Lck and are defective in BCR signaling whereas splenic B-1a cells do not express Lck and are not defective in BCR signaling (36). However, results published both before and after this study question the role of Lck in B-1 cells. An early investigation of kinase family members in peritoneal B-1a cells verified the presence of other src-kinases such as Lyn, Blk, Hck, and Syk, but not Lck (32). Subsequently Frances et al. re-examined Lck expression and found an absence of Lck in B-1a cells purified by various methods. However, despite the lack of Lck expression found in B-1a cells, defective BCR signaling was still observed (37), suggesting Lck expression does not correlate with B-1a cell hyporesponsiveness to BCR crosslinking. A few years later, it was shown by a separate group that splenic B-1a cells lacking Lck are, in fact, hyporesponsive to BCR signaling (38), unlike the finding by Dal Porto et al., which suggested splenic B-1a cells respond normally to BCR cross-linking (36). Together these studies do not support a role for Lck in the lack of NF-κB activation and proliferation by BCR-stimulated B-1 cells.

The inhibitory receptor Siglec-G has been shown to be highly expressed and functional in B cells (39). Siglec G plays an important role in B-1a cell signaling and over-expression inhibits Ca^2+^ signaling. As with SHP-1 deficiency, Siglec G deficiency enhances B-1a cell development and leads to an increase in B-1a cell number; these B-1a cells manifest enhanced signaling (39). Therefore, Siglec-G plays an important role in B-1 cell signaling and development but no clear or distinct role has been shown for Siglec G in inhibiting NF-κB activation and/or proliferation in response to BCR crosslinking.

It is important to recall the B-cell receptor complex does not function alone but is associated with additional signaling proteins, which include CD19, CD21, and CD81. These associated proteins are collectively termed the B-cell receptor co-complex, and greatly enhance the signal received after antigen binding to the BCR complex. When the BCR binds antigen coated with the complement component C3d, the complement receptor CD21 binds C3d resulting in activation of CD19 along with the BCR (40). Activation of the CD19/BCR co-complex enables B cells to respond to significantly less (10–100 fold less) antigen as compared to B cells lacking CD19 (41, 42).

CD19 is phosphorylated and activated by Lyn, a src family kinase; in turn CD19 amplifies Lyn activation and enhances activation of other src family kinase members. Subsequently Vav proteins are phosphorylated (43, 44). Lyn also serves an essential regulatory role by phosphorylation of cell surface receptors shown to negatively regulate the BCR response, such as CD22 (45). CD19 phosphorylation leads to activation of phosphatidylinositol 3-kinase (PI-3K), which phosphorylates inositol phospholipids leading to initiation of several signaling cascades via phospholipase C (PLC) and/or Ca^2+^ activation (46).

It is interesting to note B-1a cells express higher levels of CD19 than B-2 cells (47) and their development is greatly impaired in the absence of CD19 (48, 49). It has been shown BCR-induced CD19 signaling in B-1 cells is different from that of B-2 cells. In particular, following BCR engagement, B-1 cells experience shorter duration of CD19 phosphorylation and less PI-3K associated with phosphorylated CD19 (35). Both splenic and peritoneal B-1 cells overexpress Lyn and manifest impaired CD19 signaling; furthermore, Lyn inhibition allows B-1 cells to recover some responsiveness to BCR ligation (38). In addition, in Lyn^up/up^ mice that express constitutively active Lyn, splenic B-2 cells fail to proliferate in response to BCR ligation and thus acquire a signaling deficiency that parallels unmanipulated B-1 cells (50). Together these results suggest the unusual signaling characteristics of CD19 and Lyn may play an important role in the failure of B-1 cells to respond to BCR ligation. This may be exacerbated by the decreased levels of Vav1 and Vav2 in B-1 as compared to B-2 cells.

These studies over the past 20 years clearly demonstrate signaling differences between B-1a and conventional B-2 cells and point toward signaling in these two distinct subsets as being differentially regulated. However, a clear conclusion as to why B-1a cells do not activate NF-κB or proliferate in response to BCR cross-linking has not emerged. Here we synthesize a unifying hypothesis for the failure of B-1a cells to respond to BCR ligation. This hypothesis is placed in the context of the unique characteristics B-1a cells display.

## B-1a Cell Signaling through the BCR is Not Defective

The loss of NF-κB activation and proliferation in response to BCR ligation suggests B-1 cells have complete or partial loss of certain signaling pathways (deletion model). However, signaling through the BCR of B-1a cells is not completely blocked. B-1a cells activate ERK, JNK, and NF-AT in response to BCR cross-linking (26). Moreover, BCR-induced activation has also been observed in more membrane proximal mediators. Recently we have shown intact functioning of key signaling mediators by demonstrating: (1) normal phosphorylation/activation of Syk and PLCγ2 (32) after BCR ligation, and (2) a src kinase requirement for BCR-induced Syk and PLCγ2 activation (51). Furthermore, we and others have observed calcium mobilization after BCR ligation in B-1a cells, which is comparable in degree to conventional B-2 cells [data not shown and (34, 52)]. Together these results demonstrate signaling in B-1a cells is functional and capable of activating membrane proximal mediators after BCR ligation. Despite seemingly normal functioning of membrane proximal mediators after BCR ligation, the fact remains that NF-κB is not activated in B-1a cells.

**Figure 1:**
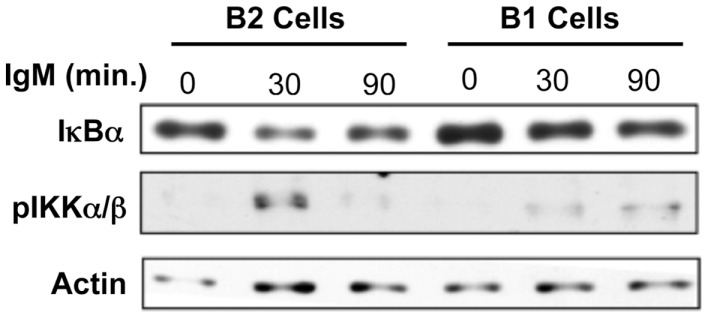
**I-kappa-B-alpha degradation and IKKalpha/beta phosphorylation analysis in response to anti-IgM in B-1a cells**. Sorted peritoneal B-1a and splenic B-2 cells were treated with anti-IgM (15 μg/ml) for the times indicated at 37°C. Afterward, the cells were washed, pelleted, lysed, and then used for western blot analysis. Results shown are representative of three independent experiments.

NF-κB activation occurs once NF-κB subunits are released from association with IκB, which allows the subunits to translocate into the nucleus (53). In previous reports the lack of NF-κB activation in response to BCR cross-linking in B-1a cells was demonstrated by an absence of nuclear expression of NF-κB components and a lack of κB-binding activity in nuclear extracts (31). However, the activation of mediators leading to NF-κB induction, such as IKKα/β phosphorylation or IκBα degradation have not been evaluated in B-1a cells after BCR ligation. Since membrane proximal signaling in B-1a cells has been shown to operate normally after BCR ligation, these more distal signaling events specific for NF-κB induction were assessed for activation status after BCR ligation. We found a large amount of phosphorylated IKKα/β protein detected at 30 min in conventional B-2 cells (Figure 1). Conversely, only a small amount of phosphorylated IKKα/β protein was detected at 30 min in B-1a cells. A dramatic decrease in IκBα protein occurs in B-2 cells after 90 min of anti-IgM stimulation (Figure 1). In contrast, only a small amount of IκBα degradation is observed in B-1a cells after 90 min of anti-IgM stimulation. These results show both phosphorylation of IKKα/β and the degradation of IκBα observed in B-1a cells is considerably less than that seen in B-2 cells stimulated with anti-IgM. Insufficient phosphorylation of IKKα/β and/or IκBα could prevent the translocation of NF-κB subunits into the nucleus. These results suggest the lack of NF-κB activation in B-1a cells after BCR ligation may originate with abnormal induction and/or regulation of phosphorylated IKKα/β and/or IκBα. If B-1a cells are still able to signal yet not able to activate NF-κB, it is possible NF-κB induction is being actively blocked, perhaps because proper phosphorylation of IKKα/β and/or IκBα is obstructed (inhibition model).

## Regulation of BCR-Induced Signaling Leading to NF-κB Activation in B-1a Cells

### Phosphatases

Our previous work emphasizes the role of phosphatases in regulating B-1a cell signaling. We examined the origin of constitutively phosphorylated ERK in B-1a cells. Despite phosphorylation of ERK, upstream signaling mediators such as Syk and PLCγ2 are not phosphorylated. However, we found after addition of tyrosine phosphatase inhibitors B-1a cells accumulated large amounts of phosphorylated Syk and PLCγ2, which did not occur with conventional B-2 cells. Moreover, this phosphorylation was blocked when B-1 cells were pre-treated with a src kinase inhibitor (51). These results suggest that signaling of upstream mediators by src kinases is taking place constitutively in B-1a cells, but rapid dephosphorylation prevents accumulation of phosphorylated intermediates. In other words, elevated phosphatase activity interferes with some, but not all, signaling events and raises the possibility that differential phosphatase expression and/or increased phosphatase activity could be playing a role in blocking anti-Ig-induced NF-κB induction in B-1a cells.

**Figure 2:**
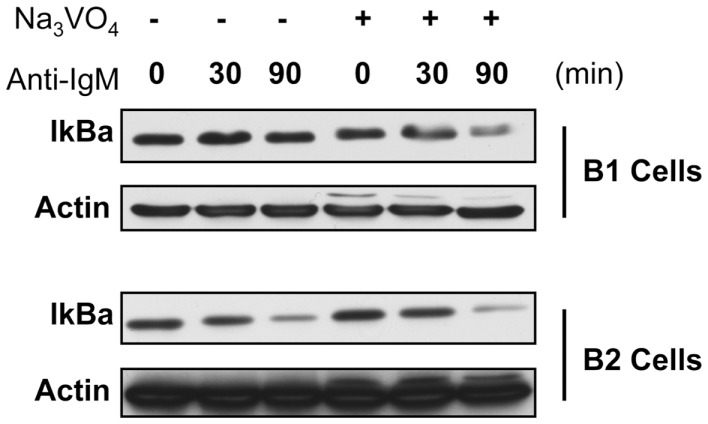
**IκBa degradation in B-1a and B2 cells in the presence or absence of phosphatase inhibition**. Sorted B-1a and B-2 cells were pre-treated for 1 h with cycloheximide (50 μM). Afterward, cells were either treated with or without anit-IgM (15 μg/ml) for 30 or 90 min, as indicated, or cells were cultured with 2 mM sodium orthovanadate (Na_3_VO_4_) for 15 min and then treated with anti-IgM for 30 or 90 min. Cells were collected, supernatants discarded, and pellets frozen at −20°C until lysed in NP-40 lysis buffer and used for western blot analysis of IκBα. Results shown are representative of two independent experiments.

To test this we assessed IκBα degradation in anti-Ig-stimulated B-1a and B-2 cells pre-treated with the tyrosine phosphatase inhibitor sodium orthovanadate. Results are presented in Figure 2. These results show in the native state, as expected, IκBα is degraded in B-2 cells after anti-Ig stimulation for 30 and 90 min, whereas little to no IκBα degradation is seen in B-1a cells similarly stimulated by anti-Ig. However, in the presence of tyrosine phosphatase inhibition, IκBα is degraded in B-1a cells stimulated with anti-Ig, just as it is in unmanipulated B-2 cells. These results strongly suggest the failure of BCR-induced NF-κB activation in B-1a cells is the result of increased tyrosine phosphatase expression and/or activity, or is the result of insufficient phosphatase inactivation.

Previous reports have attributed inhibition of NF-κB activation in other cell types to the activity of phosphatases, which is consistent with the effect of tyrosine phosphatase inhibition discussed above (Figure 2). It has been shown NF-κB activation can be affected by oxidation and/or reduction events (54, 55). Normally the cytosol is a reducing environment due to the presence of molecules such as glutathione (56), which is favorable for the activity of protein-tyrosine phosphatases (PTP). Oxidizing agents inactivate PTP by oxidizing the cysteine residue in the active site (57). Oxidizing agents, including reactive oxygen species (ROS) such as H2O2, have been shown to be produced inside lymphocytes where they regulate signaling by inhibiting PTP (55). Regardless of the stimulus, the cellular redox environment appears to play a role in the regulation of tyrosine phosphorylation events leading to activation of NF-κB (54).

The exact mechanism of how phosphatases regulate NF-κB activation is still not fully understood. It could be hypothesized that phosphatases regulate NF-κB activation by directly dephosphorylating IKKα/β thereby blocking phosphorylation of IκB proteins. Alternatively, phosphatases could act on upstream mediators specific to NF-κB activation such as NF-κB inducing kinase (NIK), which phosphorylates IKKα. An example of this type of regulation is provided in a study showing IL-1β-induced activation of NF-κB is dependent upon NIK activation, which requires ROS mediated inhibition of phosphatases for activation (58). Such studies demonstrate the role phosphatase activity and the redox environment can play in controlling NF-κB activation in response to various stimuli, which parallels the results shown here where phosphatase inhibitors cleared the way for anti-Ig-induced activation of NF-κB in B-1a cells (Figure 2). Further investigation into phosphatase expression and activity is likely to help unravel the mechanism which prevents NF-κB activation in B-1a cells in response to BCR ligation.

### HSP70

**Figure 3:**
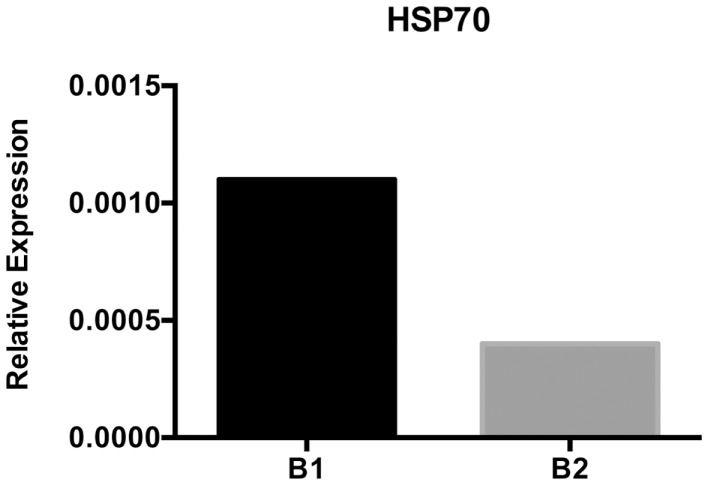
**HSP70 expression analysis**. Sorted naïve peritoneal B-1a and splenic B-2 cells were used for isolation of mRNA and subsequent cDNA synthesis after DNAse I treatment. HSP70 mRNA expression was assessed using semi-quantitative PCR. Results are representative of three independent experiments.

The HSP70 family of proteins plays a role in facilitating protein folding and preventing aggregation in the ER (Grp78/Bip) and cytosol (HSC70 and HSP70) (59). High levels of protein within cells or stress may cause an increase in HSP expression as in the case of the unfolded protein response (60, 61). B-1a cells are constantly producing natural antibody and have nearly twice as much protein per cell than B-2 cells; in keeping with this, we found levels of HSP70 gene expression are higher in B-1a cells as compared with B-2 cells (Figure 3). Interestingly, HSPs have been shown to block the NF-κB pathway through inhibition of IKK or by increasing phosphatase activity leading to a decrease in IκBα phosphorylation (62). It is feasible there is enough overall decrease in IκBα degradation produced by the increased level of HSP70 or related proteins in B-1a cells to impair NF-κB translocation to the nucleus. Other chaperones such as HSP27 and clusterin have also been shown to inhibit NF-κB activation (59); however the expression levels of these chaperones have not been examined in B-1a and B-2 cells. In addition, the expression levels of cytosolic or ER-resident HSP70 family members have not been examined. Further analysis is required to determine the site of elevated HSP70 expression in B-1a cells, because only cytosolic HSP70 would result in inhibition of NF-κB. HSP70 remains an important candidate for modulation of B-1a cell NF-κB induction.

### IL-10

B-1 cells have long been associated with IL-10 expression and secretion, and although B-1a and B-2 cells secrete IL-10 after antigenic stimulation (27), only B-1 cells secrete IL-10 spontaneously (24). Interestingly, it has been shown that IL-10 inhibits TNF-induced activation of NF-κB by preventing IKK activity and DNA binding of the NF-κB subunits (63). Further, IL-10 as well as IL-2 and IL-5 have been shown to regulate expression of certain phosphatases (64, 65). Therefore, it is possible that induced and/or constitutively secreted IL-10 in B-1 cells contributes to differences in phosphatase expression and/or activity in B-1a cells as compared to B-2 cells.

## Discussion

BCR mediated signaling in B-1a cells functions normally in terms of phosphorylation of membrane proximal mediators such as PLCγ2 (51), Syk (51), and Ca2+ mobilization (34). While these findings illustrate functional membrane proximal BCR signaling, IKKαβ phosphorylation and IκBα degradation in response to BCR stimulation is impaired in B-1a cells (Figure 1). However, IκBα degradation is rescued if B-1a cells are pre-treated with the tyrosine phosphatase inhibitor, sodium orthovanadate (Figure 2). Collectively, these results suggest signaling in B-1a cells is differentially regulated by phosphatases, as compared to B-2 cells.

**Figure 4:**
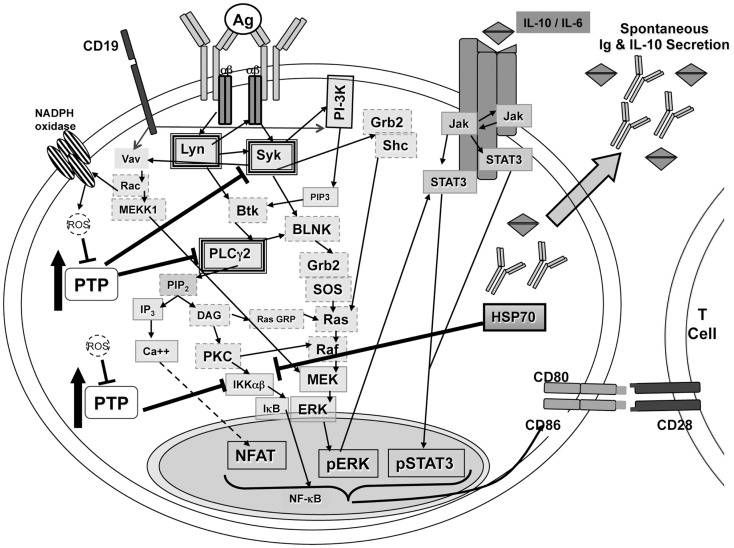
**Signaling in B-1a cells**. B-1a cells constitutively secrete IgM and IL-10 without prior stimulation. In addition, B-1a cells have constitutive levels of activated ERK, STAT3, and NF-AT, yet are not able to activate NF-κB in response to BCR ligation. It has been shown constitutive ERK activation in B-1 cells is the result of chronic signaling through the BCR (51). The constitutive ERK activation was shown to be dependent upon src kinases, PI-3K, Syk, and PLCγ2, which are heavily outlined in the illustration. Inhibition of PI-3K or Syk also blocked the constitutive levels of CD86 on B-1a cells, which is known to play an essential role during allogeneic stimulation of T cells (23, 51). All mediators outlined in a dashed gray line may play a role in signal transduction leading to constitutive ERK activation but have not been tested. Phosphatase activity, denoted as PTP, has been shown to control phosphorylation of Syk and PLCγ2 differentially in B-1a cells as compared to B2 cells (51). In addition, inhibition of phosphatase activity in B-1 cells was shown, Figure 2, to allow IκBα degradation in B-1a cells after BCR ligation. Therefore, PTP activity in B-1a cells inhibits NF-κB activation by an unknown mechanism. It is hypothesized that this mechanism involves HSP70 and IL-10, which were both shown to be expressed at a higher level in naive B-1a cells as compared to naive B2 cells, Figure 3 and Ref. (24). Furthermore, it has been shown that inhibition of constitutively active Lyn allowed for partial recovery of B-1a cells’ responsiveness to BCR ligation (38). In addition, suboptimal Vav levels in B-1a cells (35) may not be sufficient for the production of ROS, which are necessary to inhibit phosphatases to allow activation of NF-κB.

Phosphatase activity has been shown to play a role in regulating the activation of NF-κB (54, 58). Reduction/oxidation events within the cell, particularly generation of ROS, can regulate phosphatase activity (55, 57). If high levels of phosphatases are controlling BCR-induced signaling in B-1a cells and ROS play a role in controlling phosphatases, two questions arise: (1) what regulates expression of phosphatases in B-1a cells; and, (2) how do signals derived from LPS and CD40L generate enough ROS, or bypass the need for ROS, to allow NF-κB activation whereas BCR ligation fails to do so? The unique characteristics of B-1a cells (summarized in Figure 4) may relate to these issues.

First, HSP70 and IL-10 can both play a role in influencing the expression of phosphatases (62–67). Here we demonstrated HSP70 is highly expressed in naïve B-1a cells as compared to B-2 cells (Figure 3). HSP70 has been shown to inhibit NF-κB signaling by inducing phosphatase activity and by directly interacting with IKKγ, thereby disrupting the IKKγ protein from binding to the IKKαβ complex, which renders it inactive (62, 66–68). It is possible one or both of these mechanisms operates in B-1a cells thereby blocking NF-κB activation after BCR ligation. However, the over-expression of HSP70 may merely reflect an increased need for chaperones in B-1a cells due to their continuous secretion of IgM. Furthermore, IL-10 is expressed in naïve B-1a cells but not in naïve B-2 cells (24). IL-10 has also been shown to inhibit NF-κB activation by preventing IKK activity (63) and has been shown to regulate the expression of certain phosphatases (64, 65). These are just two examples of how phosphatases might be differentially regulated in B-1a cells as compared to B-2 cells.

Secondly, the reason for lack of NF-κB activation in response to BCR ligation yet normal activation in response to CD40L or LPS may lie in the difference in signal strength delivered by these different stimuli. Perhaps a stronger signal is required, which may come from activation of receptors such as a toll-like receptor (TLR) or CD40. This type of additional signal requirement for activation of lymphocytes via inactivation of phosphatases has been previously suggested for B-2 cells (55). Further, it has been demonstrated B-1 cells proliferate in response to BCR ligation if the receptors are hyper-crosslinked (35). It may be that B-1a cells are relatively “exhausted” through tachyphylaxis as a result of chronic signaling (51) and thus require an unusually strong signal when delivered through the BCR as opposed to TLR or CD40. The high levels of constitutively active Lyn in B-1a cells may also be responsible for the requirement of an unusually strong signal through the BCR as the constitutively active Lyn might be activating inhibitory receptors such as SHP-1 and SHIP-1 (38).

We propose a certain level of phosphatase inactivation by ROS is required for full B-1 cell activation to occur. BCR cross-linking can cause an increase in ROS production, which can inactivate phosphatases thereby allowing signaling to propagate through the cell. After BCR ligation in B-1a cells NF-κB is not activated. Therefore, we hypothesize phosphatase activity must not be appropriately inhibited after BCR ligation to allow for NF-κB activation. Normally, ROS are generated by NADPH oxidase, which is activated by signal transduction via Rac or PKC (55, 69). Rac is a GTPase activated in B cells via the guanine nucleotide exchange factor Vav (70). Vav is recruited by CD19 and phosphorylated by Lyn (43). A few years ago it was reported the lack of proliferation to BCR cross-linking by B-1 cells is the result of defective CD19 signaling, which is due to a reduced level of Vav proteins in B-1 cells (35). The essential role of the Vav proteins is further demonstrated in B-2 cells deficient in Vav, which show no activation of NF-κB in response to BCR ligation (35). These results combined with the results presented here and Reth’s model of lymphocyte activation (55) suggest there is a lack of sufficient levels of Vav proteins in B-1 cells to activate Rac. Insufficient Rac activation would not allow for necessary ROS production through NADPH oxidase to overcome the phosphatase activity regulating NF-κB components. Therefore, a second signal or stronger signal is necessary in B-1 cells for phosphatase inactivation.

## Hypothesis

**Figure 5:**
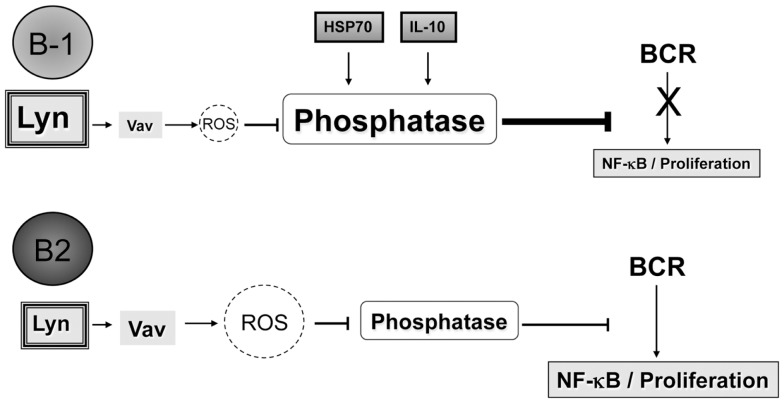
**Differences in Vav proteins, IL-10, and/or HSP70 expression between B-1a and B2 cells lead to the inability of B-1a cells to activate NF-κB and proliferate after BCR ligation**. It is hypothesized that B-1a cells have increased levels or differential expression of phosphatases as compared to B2 cells, which could be at least partially the result of the increased basal expression of IL-10 and/or HSP70 seen in B-1a cells but not B2 cells. Furthermore, suboptimal Vav levels in B-1 cells (35) may not be sufficient for the production of ROS, which are necessary to inhibit phosphatases to allow activation of NF-κB after BCR ligation. Over-expression of the src kinase Lyn may also play a role in the non-response of B-1a cells to BCR ligation, perhaps by phosphorylation of inhibitory receptors. The mechanism through which Lyn contributes to B-1a cell non-responsiveness to BCR ligation is not clear; however, it has been demonstrated blocking Lyn activity can allow B-1a cells to respond to respond to BCR crosslinking.

In synthesizing previous reports and new data presented here, we suggest a new hypothesis for the lack of NF-κB activation and proliferation in B-1a cells after BCR ligation. We summarize this hypothesis in Figure 5.

B-1a cells are unable to activate NF-κB or proliferate after BCR cross-linking due to increased phosphatase abundance and/or activity, which could be a result of basal expression of IL-10 and/or HSP70. The increased phosphatase abundance and/or activity is not able to be overcome by BCR ligation alone due to constitutively active Lyn and/or a lack of Vav protein expression in B-1 cells, which does not allow for proper production of ROS needed to inhibit the phosphatases present in B-1 cells.

## Future Directions

A remaining question revolves around the nature of the phosphatase activity opposing BCR signaling in B-1a cells. Signaling deficiencies in antigen-experienced B cells, including germinal center B cells (71) and anergic B cells (72) have been attributed to SHP-1 and SHIP-1 respectively. Like anergic B cells (which express low levels of CD5) B-1a cells are thought to be antigen-experienced, so SHP-1 and SHIP-1 are candidates for BCR regulation here as well, although other possibilities, such as DUSP phosphatases, may be involved. Although elucidating the source of B-1a cell phosphatase activity is important, similar attention should focus on understanding the way in which phosphatase activity is normally overcome, a process that fails in B-1a cells.

## Conflict of Interest Statement

The authors declare that the research was conducted in the absence of any commercial or financial relationships that could be construed as a potential conflict of interest.
